# RNA-Sequencing approach for exploring the therapeutic effect of umbilical cord mesenchymal stem/stromal cells on lipopolysaccharide-induced acute lung injury

**DOI:** 10.3389/fimmu.2022.1021102

**Published:** 2022-10-20

**Authors:** Enhai Cui, Luwen Zhang, Xin Pan, Qiang Zhang, Ling Zhang, Feifei Wu, Na Chen, Lu Lv, Wenyan Chen, Hong Chen, Aifu Lin, Feng Wang, Jinfeng Liang, Ruolang Pan

**Affiliations:** ^1^ Department of Huzhou Central Hospital, Affiliated Huzhou Hospital of Zhejiang University School of Medicine, Hangzhou, China; ^2^ Institute of Genetics, Zhejiang University School of Medicine, Hangzhou, China; ^3^ Institute for Cell-Based Drug Development of Zhejiang Province, S-Evans Biosciences, Hangzhou, China; ^4^ Institute for Cell-Based Drug Development of Zhejiang Province, Key Laboratory of Cell-Based Drug and Applied Technology Development in Zhejiang Province, Hangzhou, China; ^5^ College of Life Sciences, Zhejiang University, Hangzhou, China; ^6^ Department of Nephrology, Key Laboratory of Kidney Disease Prevention and Control Technology, Hangzhou Traditional Chinese Medicine (TCM) Hospital Affiliated to Zhejiang Chinese Medical University, Hangzhou, China; ^7^ Department of Drug Evaluation, Zhejiang Center for Drug & Cosmetic Evaluation, Hangzhou, China

**Keywords:** acute lung injury, umbilical cord mesenchymal stem/stromal cells, gene ontology annotation, Kyoto Encyclopedia of Genes and Genomes enrichment, protein-protein interaction network identification

## Abstract

Acute lung injury (ALI) is significantly associated with morbidity and mortality in patients with critical diseases. In recent years, studies have identified that mesenchymal stem/stromal cells (MSCs) ameliorate ALI and pulmonary fibrosis. However, the mechanism underlying this outcome in ALI has not yet been investigated. In this study, RNA sequencing technology was used to analyze the gene expression profile of lung tissue in lipopolysaccharide (LPS)-induced ALI rats following treatment with human umbilical cord MSC (HUCMSC). Differential expression analyses, gene ontology annotation, Kyoto Encyclopedia of Genes and Genomes enrichment, protein–protein interaction network identification, and hub gene analysis were also performed. HUCMSC treatment decreased inflammatory factor production and alveolar exudates, and attenuated lung damage in LPS-induced ALI rats. The RNA-Seq data indicated that HUCMSC treatment activated the IL-17, JAK-STAT, NF-κB, and TNF-α signaling pathways, increased oxygen transport, and decreased extracellular matrix organization. HUCMSC exert beneficial effects on ALI *via* these signaling pathways by reducing inflammation, inhibiting pulmonary fibrosis, and improving lung ventilation. Moreover, our study further revealed the hub genes (*Tbx2*, *Nkx2-1*, and *Atf5*) and signaling pathways involved in HUCMSC treatment, thus providing novel perspectives for future research into the molecular mechanisms underlying cell treatment of ALI. HUCMSC can regulate multiple genes and signaling pathways, which can prevent LPS-induced lung damage in an ALI rat model.

## Introduction

Acute lung injury (ALI) is a common and serious illness with a high mortality rate. Clinically, it usually manifests as alveolar and lung parenchymal inflammation, and involves alveolar-capillary membrane damage, local neutrophil accumulation, and an imbalance of pro- to anti-inflammatory factors in the lungs. ALI can develop into a severe form called acute respiratory distress syndrome (ARDS) (1). ARDS increases the permeability of pulmonary vascular endothelial cells and weight of lung tissues, and reduces the number of ventilated alveoli. It is difficult to treat and has a high mortality rate. Although more than 3 million people develop ARDS every year worldwide, accounting for 10% of patients in intensive care, there is currently no standardized treatment method (2, 3). The current clinical treatment includes only supportive therapy, Chinese and Western medicine, and auxiliary ventilation support. However, these treatments are ineffective and do not decrease the mortality rate (4). Therefore, there is an urgent need to identify effective treatments to improve ALI prognosis.

ALI experimental animal models can be established by a variety of substances, such as lipopolysaccharide (LPS) (5), oleic acid (6), Tween (7), among which LPS is the most widely used. LPS is derived from the cell wall of Gram-negative bacteria. LPS can induce ALI animal models in a short time by inhalation or intravenous administration. LPS can cause microvascular lung injury after exposure in animals, resulting in direct (pneumonia) and indirect lung injury (sepsis). It is very similar between LPS-induced lung injury and human ALI/ARDS. Therefore, this experiment established a rat model of ALI induced by inhalation of LPS.

Mesenchymal stem/stromal cells (MSCs) are adult stem cells derived from the mesoderm, which can be obtained from various tissues, including the bone marrow, adipose tissue, placenta, and umbilical cord (8). There has been increasing research on the characteristics and biological functions of MSCs, and studies have shown that MSC transplantation can significantly improve the repair of damaged tissue, including bone defects, brain injury, myocardial infarction, and acute liver and lung injuries. MSCs showed benefits in lung injury, such as alleviation of lung inflammation, inhibition of pulmonary edema development, amelioration of lung injury, and reduction of animal mortality rate (9). Wang Fengyun (10) reported that MSCs can reduce the lung pathological injury, regulate the immune balance and promote lung regeneration in ALI animals caused by LPS or bacteria. Studies have shown that usage of MSC in ALI animals by intratracheal delivery can significantly reduce pulmonary edema and improve alveolar epithelial permeability (11, 12). Human umbilical cord MSC (HUCMSC) are easy to isolate, culture, and amplify, with no restrictions on moral and ethical issues (13). HUCMSC transplantation can ameliorate motor function, tissue repairing and reducing apoptosis in spinal cord injury (14). It is also showed that HUCMSC transplantation may be a novel therapeutic approach for treating acute radiation injury (15), traumatic brain injury (16), acute kidney injury (17), radial nerve injury (18). In addition, researcher has demonstrated the therapeutic effects of HUCMSC in ALI models (19). However, the mechanisms underlying the beneficial effects of MSCs on ALI have not yet been elucidated.

We hypothesize that there is a complex cell and cytokine network in ALI that results in changes in the expression of several genes. Therefore, we aimed to investigate the mRNA expression profiles in lung tissues from rats with LPS-induced ALI with or without MSC treatment. We investigated the mechanism of HUCMSC-treated ALI and provided theoretical and data support for further clinical investigations.

## Materials and methods

### Animals

Healthy pathogen-free Sprague-Dawley rats (male, 12 weeks old) were provided by Shanghai Silaike Experimental Animal Co. and reared at 22 ± 2°C under a 12 h light/dark cycle. The humidity was maintained at 50%–60%. All experiments were conducted according to the protocols approved by the local Medical Animal Experiment Ethics Committee.

### Isolation and culture of HUCMSC

HUCMSC were isolated and characterized as previously described (9). Briefly, umbilical cord tissues from healthy women were washed three times and cut into approximately 1 mm^3^ pieces. The pieces evenly spread on the bottom of a 75cm^2^ flask with the serum-free MSC culture medium (TBD Science, China) for primary adherent culture and cultured in a 5% CO_2_ incubator at 37°C. The medium was refreshed every 3-4 days. After achieving 80%–90% confluence, the cells were digested with 0.25% trypsin-EDTA (Gibco, Carlsbad, CA, USA) 2 minutes for passaging. The cells were seeded into 175cm^2^ flasks at a density of 6000-8000 cells/cm^2^. Until the cell confluence reached 80%-90%, the cells were digested and collected to obtain the passage one of HUCMSC. Repeat the above operations for subculture. HUCMSC at passage five were characterized by morphology, mesenchymal lineage differentiation and surface marker expression. The capacity of osteogenic, adipogenic, and chondrogenic mesenchymal lineage differentiation was detected using Alizarin Red, Oil Red O, and Alcian Blue staining (OriCell, Guangzhou, China), respectively. Surface marker expression was also characterized by flow cytometry with CD73, CD90, CD105, CD34, CD14 and HLA-DR antibodies (BD Bioscience, San Jose, CA, USA) as described by previous study (20). Then the cells were used for further experiments.

### Establishment of an LPS-induced ALI rat model and HUCMSC treatment

SD rats were randomly divided into three groups: the normal control group (Normal), ALI group (ALI), and HUCMSC-treated group (HUCMSC) (n=9). The ALI model was established by LPS inhalation. Briefly, the animals were anesthetized with 3% pentobarbital (50 mg/kg body weight, Sigma, USA) and intratracheally nebulized with 5 mg/kg LPS (*Escherichia coli* O55:B5; Sigma, CA, USA) on day 1 and with 1 mg/kg on day 3. HUCMSC were transplanted after 3 h of LPS stimulation on days 1 and 3 using tail vein injection. According to the results of our preliminary experiments, a dose of 0.4 × 10^6^ cells/kg for HUCMSC was used. On day 4, the animals were sacrificed by an overdose of pentobarbital (150 mg/kg) intraperitoneally in accordance with a previous study (21). Arterial blood, abdominal main vein, bronchoalveolar lavage fluid and lung samples were collected for further analysis.

### Evaluation of therapeutic effect of HUCMSC

On day 4, the rats (n=6) were anesthetized and assessed to determine the therapeutic effect of HUCMSC. Arterial blood samples were collected (approximately 500 μL) from rats and analyzed using a blood gas detection instrument (Radiometer ABL700). Two milliliters of serum from the abdominal main vein was collected, and TNF-α and IL-1β levels were determined using an enzyme-linked immunosorbent assay kit, according to the manufacturer’s instructions (mlbio). Bronchoalveolar lavage fluid was prepared according to a previously described method [10]. The lavage fluid was collected and centrifuged at 2000 rpm at 4°C for 10 min. The supernatant was tested for total protein concentration using a BCA assay kit (Beyotime Institute of Biotechnology) according to the manufacturer’s instructions. The sediment was resuspended in 1 mL of phosphate-buffered saline buffer to measure the number of neutrophils, lymphocytes, and total white blood cells using an automatic blood cell analyzer (Countstar IC1000). The lung tissues were harvested for histological analysis. Lung tissue samples were fixed in 4% paraformaldehyde solution (Sangon Biotech), embedded in paraffin blocks, and sliced into multiple 5 μm-thick sections. The sections were stained with hematoxylin and eosin (H&E; Sangon Biotech), photographed under a light microscope (Carl Zeiss).

### RNA isolation and sequencing

Total RNA was extracted from lung tissues of SD rats (n=3) using TRIzol reagent (Invitrogen), according to the manufacturer’s instructions. The purity and integrity of RNA was analyzed using an Agilent Bioanalyzer 2100 (Agilent Technologies). RNA sequencing was performed using the Illumina HiSeq 4000 sequencing system. The MapSplice program was used for RNA-Seq data mapping. Transcripts per kilobase per million was used to measure the expression levels of each gene. Use fastqc next-generation sequencing data quality analysis software to analyze the quality of paired-end data fastq (.gz) files. Use hisat2 to align the filtered fastq.qz data file (with the cDNA sequence inside) to the corresponding reference genome to generate the corresponding sam file.

### Assembly screening for differentially expressed genes

Differentially expressed genes (DEGs) were identified using DESeq2 package. Obtain the original Count value, and then use DEseq2 to obtain the differential genes between groups. Use dds1<- dds1[rowSums(counts(dds1)) > 1], to filter out low abundance data. condition <- factor[c(“A”,”A”,”A”,”B”,”B”,”B”,”C”,”C”,”C”)], between the three groups The between-group comparisons are contrast=c (“condition”, “B”,”A”), contrast=c (“condition”, “C”,”B”) and contrast=c (“condition”, “C”, “A”). After the comparison, the differential gene table is exported, **supplementary data** such as **Supplementary DEG.csv, DEGs KEGG.csv and DEGs BP.csv**. The significance of differences in gene expression was bound using a false discovery rate of <0.05 and an absolute fold change of >1.5. Volcano plots and heat maps were generated using OmicShare online tools (http://www.omicshare.com/tools) (R package: cluster profiler). Venn diagrams were developed using the online tool Draw Venn Diagrams (http://bioinformatics.psb.ugent.be/webtools/Venn/).

### Gene ontology annotation and Kyoto Encyclopedia of Genes and Genomes enrichment

Gene ontology (GO) classification of DEGs and distribution analysis of gene function in species at the macrolevel were conducted using WEGO software (22), whereas pathway enrichment analysis was performed using the Kyoto Encyclopedia of Genes and Genomes (KEGG) database (http://www.genome.jp.kegg/) (23). GO categories with an adjusted p-value <0.05 were regarded as enriched by DEGs. The results of multigroup enrichment analysis were displayed using the R package: cluster profiler (compareCluster).

### Protein–protein interaction network and hub gene analysis

A protein–protein interaction network was constructed using the Search Tool for the Retrieval of Interacting Genes (STRING, http://www.string‐db.org/) and viewed using Cytoscape (3.8.0) (24). In database search, the species was set to ‘Rattus norregicus,’ the confidence score cutoff was set at 0.4, and other settings were set to default. Use the Cytoscape app’s plugin cytoHubba to calculate the top 20 degree genes in each interaction network.

### Statistical analysis

All experiments were conducted in triplicate. Data are presented as the mean ± standard deviation. Statistical significance was determined using Student’s *t*-test (unpaired). Statistical analysis was performed using GraphPad Prism 6.0 software, and *P* < 0.05 was considered to indicate a statistically significant difference.

## Results

### HUCMSC reduce LPS-induced ALI symptoms and damage in rats

**Figure 1:**
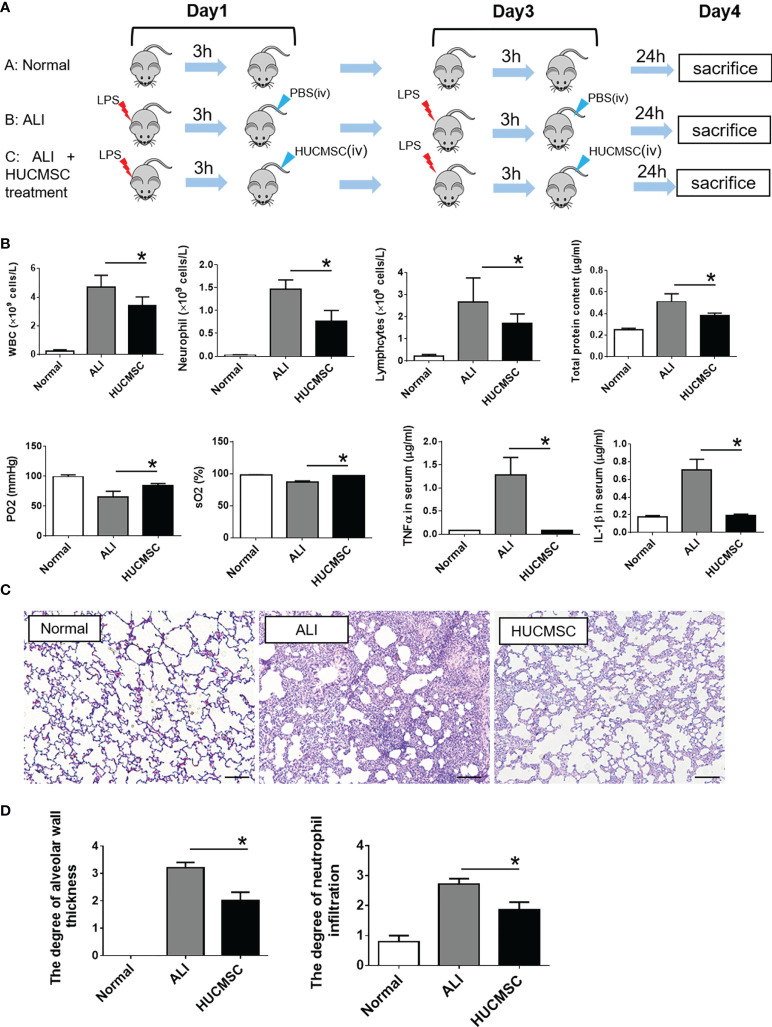
Evaluation of therapeutic effect of HUCMSC on LPS-induced ALI rat model. **(A)** Flow diagram of the experiment; **(B)** The determination of cell components in BALF, arterial blood gas parameters, and inflammatory cytokines in serum; **(C)** Representative images of histopathologic changes in different treatment groups; **(D)** Pathological scores of alveolar wall thickness and neutrophil infiltrations. Normal, normal group; ALI, LPS-induced ALI group; HUCMSC, LPS-induced ALI group with HUCMSC treatment. n=6. Scale Bars=50μm. *P < 0.05.

HUCMSC were characterized (**Supplementary Figure 1**) and used to treat LPS-induced ALI rats *via* tail vein injection (**Figure 1A**), and the treatment effects were evaluated. As depicted in **Figure 1B**, ALI caused a significant increase in white blood cells, neutrophils, lymphocytes, and total protein concentrations, which represented augmented endothelial and epithelial permeability. However, HUCMSC treatment significantly reduced these increases (*P* < 0.05). The results of arterial blood gas analyses showed that the decrease in oxygen pressure and oxygen saturation caused by ALI could be counteracted with HUCMSC treatment, suggesting that pulmonary ventilation was restored. Compared to the ALI group, the HUCMSC group showed marked decrease in the expression levels of the inflammatory cytokines TNF-α and IL-1β, indicating that lung inflammation was reduced by HUCMSC. Analogously, the pathological results also demonstrated that HUCMSC treatment significantly decreased alveolar wall thickness and extent of neutrophil infiltration (*P* < 0.05) (**Figures 1C, D**). These results demonstrate that HUCMSC can inhibit and attenuate ALI occurrence and development, which is consistent with previous studies.

### Transcriptomic profiles and differential expression analysis

**Figure 2:**
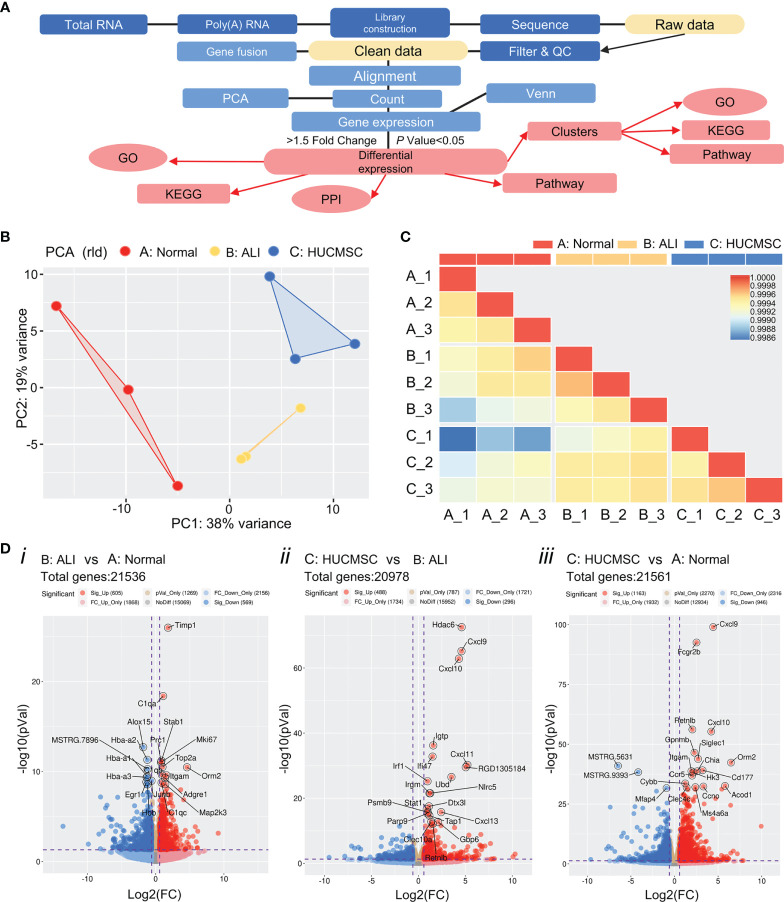
The global gene expression profiles were examined using RNA-seq. **(A)** Overview of the main steps for RNA-Seq data analysis. **(B)** Principal component analysis (PCA) of the RNA-Seq data from the samples in three different group. The same color represents different replicates of the same stage. **(C)** Heat maps showing the correlation ship between three groups. **(D)** Volcano plot of differentially expressed genes. Blue dots indicate down-regulated genes, red dots represent genes that were up-regulated expressed and yellow points show the significant differential genes (p values < 0.05 determined by DESeq2). A, normal group; B, LPS-induced ALI group; C, LPS-induced ALI group with HUCMSC treatment. n = 3.

The gene expression profile of lung tissue was examined using RNA-Seq, according to the process shown in **Figure 2A**. Lung tissues from the different groups were dissociated prior to RNA-Seq processing. RNA-Seq data mapping was accessed *via* the MapSplice program, and a total of 30000 genes were identified. The percent of reads mapped to genome regions of each sample are shown in **Supplementary Figure 2**. Principal component analysis revealed that the treatment groups were separated into three distinct cell clusters (**Figure 2B**). The correlation coefficients were all >0.85 according to the heatmap analysis, suggesting that the samples in the same group are concordant (**Figure 2C**). Raw data were collected to carry out standardized treatment and the genes were compared between A: Normal, B: ALI and C: HUCMSC groups (**Supplementary Figures 3A–C**). High sample integrity showed in the three group samples (**Supplementary Figure 3D**). These results demonstrate that the RNA-Seq data are reliable and could be used for further studies. Use R packages (ggplot2 and ggrepel) to visualize the comparison results between groups, draw volcano plots and label the number of genes and the number of up-regulated genes with differences, as well as label the top 20 genes with significant differences between groups according to the *P* value (**Figure 2D**). The results showed that 605 genes were significantly up-regulated and 569 genes were significantly down-regulated as ALI vs Normal group (**Figure 2Di**). 488 genes were significantly up-regulated and 296 genes were significantly down-regulated as HUCMSC vs ALI group (**Figure 2Dii**). 1163 genes were significantly up-regulated and 946 genes were significantly down-regulated as HUCMSC vs Normal group (**Figure 2Diii**).

**Figure 3:**
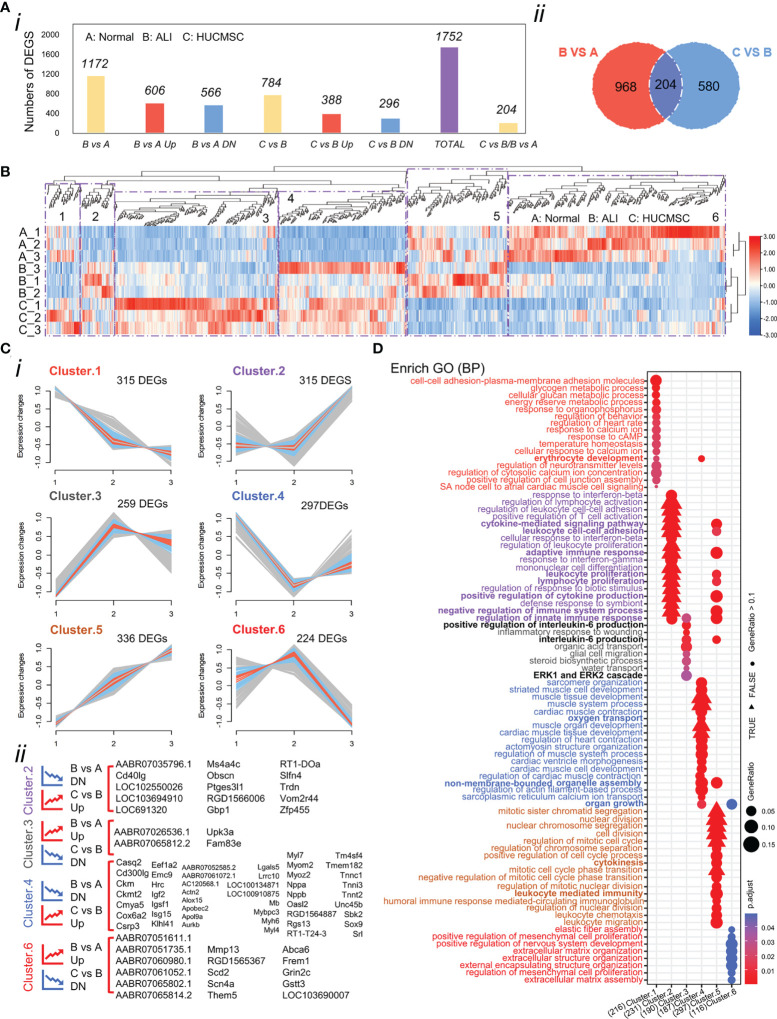
Analysis of the differentially expressed genes (DEGs). **(A)** Number of DEGs in different experimental groups. **(i)**The number of DEGs was determined by DESeq2 (|fold changes| > 1.5; P < 0.05). (ii)Venn diagram shows the different genes between B vs A and C vs **(B)** (as determined by DESeq2; P < 0.05). **(B)** Heatmap of the 1752 DEGs in the three groups. Colors represent the normalized gene expression values of DEGs (TPM). **(C)** Clustering the DEGs reveals 6 clusters, the expression trend of which were demonstrated. Blue and red colored lines correspond to genes with high membership value. Each square represents a profile of gene expression trend. **(i)** Number of differential genes under each patter. **(ii)** Genes under trend fall under pattern 2,3,4, and 6. The graph shows differentially expressed genes that fit the trend requirements under these four patterns. **(D)** Overrepresented biological process gene ontology (GO) terms in 6 clusters. Biological process GO terms associated with highly expressed genes in each cluster. Each GO term is denoted by a bubble. The color intensity of each bubble indicates p-adjust value of the corresponding GO term, and the size corresponds to the ratio of queried genes in the gene set associated with a given GO term. The GO terms enriched in each cluster are color-coded by cluster. A, normal group; B, LPS-induced ALI group; C, LPS-induced ALI group with HUCMSC treatment. n = 3.

First, we identified genes that were consistently differentially expressed in lung tissues between ALI and normal rats. A total of 1,172 genes were identified as significantly differentially expressed, of which 606 (51.7%) genes were upregulated and 566 (48.3%) genes were downregulated (**Figure 3A**). Differential expression analysis between the ALI and HUCMSC groups revealed 784 DEGs, of which 388 were upregulated and 296 were downregulated (**Figure 3A**). Among these DEGs, 29 were upregulated in ALI but downregulated in the HUCMSC group. The common significantly different gene lists are shown in **Supplementary Table 1**. Downregulation of seventy-nine genes in ALI was offset by upregulation following HUCMSC injection, suggesting that these DEGs may be relevant to HUCMSC treatment. To include the highest confidence genes in the cluster analysis, 1,752 DEGs were selected based on the highest coefficient of variation in the total dataset. The differential gene clustering heatmap is shown in **Figure 3B**, and analysis the differential genes using the R package: Mfuzz (Normalized as in **Supplementary Figure 4**). As shown in **Figures 3B, C**, we identified six clusters, and the DEGs in clusters 2, 3, 4, and 6 exhibited the trend that the expression change induced by LPS could be counteracted by HUCMSC.

### GO analysis

**Figure 4:**
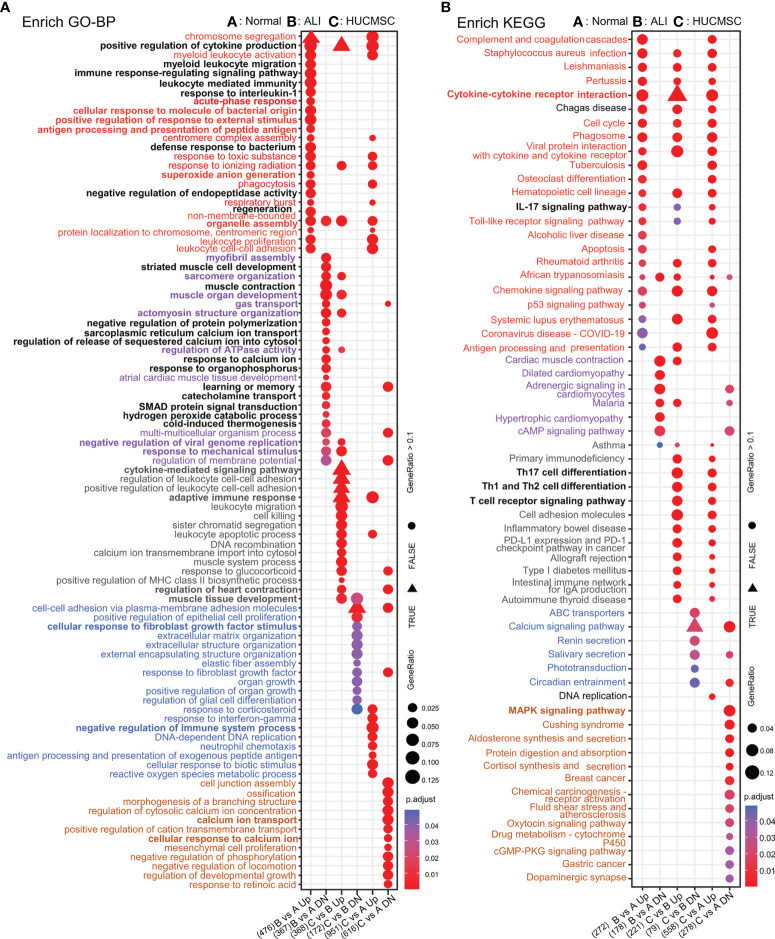
The GO/KEGG enrichment analysis results of DEGs between groups. **(A)** GO analysis of DEGs that were upregulated and downregulated in 6 groups. **(B)** KEGG analysis of DEGs that were upregulated and downregulated in 6 groups. Adjusted *P* value represents significance of terms; GeneRatio represents the ratio of the number of DEGs targets in this GO/KEGG term to the number of DEGs targets in all GO/KEGG term. GO, gene ontology; KEGG, Kyoto Encyclopedia of Genes and Genomes; DEGs, differentially expressed genes.

Differences in gene expression can contribute to phenotypic variations. GO enrichment analysis was performed based on DEG results for the functional annotation of transcriptome data. As depicted in **Figure 3D**, the enriched GO terms were identified and distributed into distinct biological processes, which did not overlap between the six clusters (**Supplementary Figure 4A**). we interestingly found that cluster 3 and 5 with different trends were linked through the biological process of IL6 production, and cluster 5 affected the positive regulation of IL6 production in cluster 3 through genes (Il1a/Tlr8/Tlr2 etc) in this process, and adaptive immune respose/cytokine-mediated signaling pathway biological processes indicate close connections between different clusters (**Supplementary Figure 4B** and **Figure 3D**).The GO terms of Cluster 2 were categorized into regulation of leukocytes, T cells, and lymphocytes. The GO terms of cluster 4 were categorized into regulation of oxygen transport, muscle contraction, and cardiac muscle development. The decreased expression of the two DEG clusters in ALI rats could be offset by HUCMSC treatment. The GO terms of clusters 3 and 6 were related to processes involved in inflammation and cellular function, respectively, including the production and regulation of IL-6, glial cell migration, elastic fiber assembly, MSC proliferation, and extracellular matrix (ECM) organization. The DEGs in these two clusters were more highly expressed in the ALI group but repressed by HUCMSC treatment (**Figure 3D**). MA plot showed the top40 hub genes between normal, ALI and HUCMSC groups (**Supplementary Figures 5A, B**) and qPCR with specific primers (**Supplementary Primers**) was carried out to validate the expression of several candidate DEGs (**Supplementary Figures 5C, D**). GO enrichment analysis was also performed after grouping genes that increased or decreased in different groups (**Figure 4A**). For the supplementary data between the LISTS of DEGs between groups and GO enrichment results, please refer to the files **Supplementary DEGs.csv** and **Supplementary DEGs BP.csv**.

### KEGG analysis

**Figure 5:**
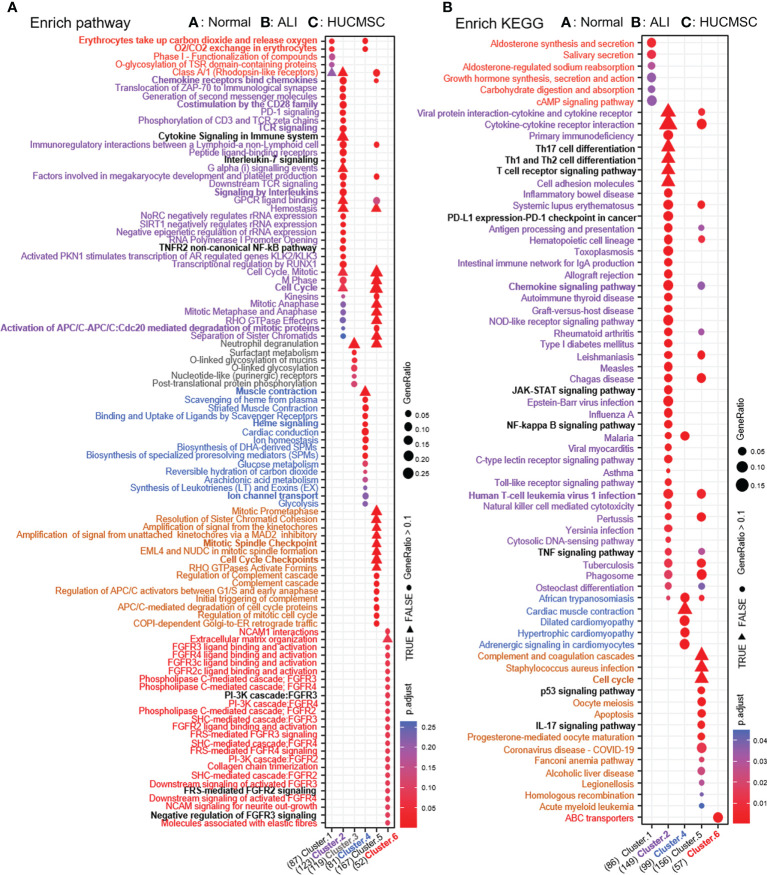
Pathway enrichment analysis. **(A)** Enrich pathway results of six clusters. **(B)** KEGG enrichment analysis by Kobas3.0 platform. The depth of color represents the size of adjust-P value. The size of the ball represents the ratio of genes enriched in the pathway.

To examine the differences in gene pathways, KEGG enrichment analysis was performed using R package: clusterprofiler (compareCluster); the pathways are shown in a bubble graph. As depicted in **Figure 5A**, some pathways were inhibited in the ALI group and activated in the HUCMSC group. The KEGG and pathway enrichment results of the concerned cluster 2, 3, 4, and 6 genes show that Th17, Th1, and Th2 cell differentiation, T cell receptor signaling pathway, PD-L1 expression, PD-1 checkpoint, the JAK-STAT, NF-κB, TNF-α, and Toll-like receptor signaling pathways (**Figure 5B**). The pathways promoted in ALI but suppressed by HUCMSC were mainly enriched in neuroactive ligand-receptor interaction, phenylalanine metabolism, the Hippo, calcium, PI3K, and IL-17 signaling pathways. Moreover, the DEGs in cluster 5, which were mainly associated with cell proliferation and apoptosis, was continuously activated in the HUCMSC group, higher expression levels than that in the ALI group. Similarly, KEGG enrichment analysis was performed after grouping genes that increased or decreased in different groups (**Figure 4B**) and the hub gene enrich results of KEGG also show in **Supplementary Figure 6B**. For the supplementary data between the LISTS of DEGs between groups and their KEGG enrichment results, please refer to the files **Supplementary DEGs.csv, DEGs KEGG.csv and DEGs BP.csv**.

### Protein–protein interaction analysis and hub gene analysis

**Figure 6:**
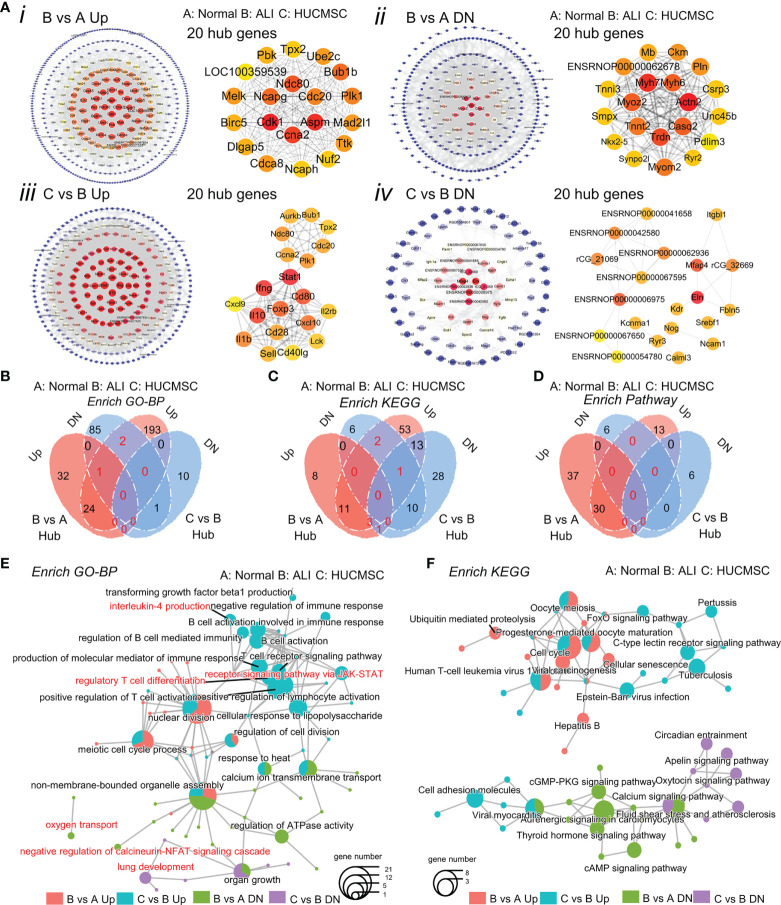
Analysis of hub genes. **(A)** Construction of the PPI network and Verification of hub genes. Top 20 hub genes are selected with the tool STRING and cytoscape. **(B-D)** Venn diagram shows the up-regulated and down-regulated genes in GO enrichment **(B)**, KEGG enrichment **(C)** and enrich pathway **(D)**. **(E, F)** The cnetplot shows the connection in the GO terms **(E)** and KEGG pathways **(F)**. **(A)**, normal group; **(B)**, LPS-induced ALI group; **(C)**, LPS-induced ALI group with HUCMSC treatment. (n = 3).

To analyze the molecular relationship between the implicated genes and proteins, PPI analysis was performed using the STRING database. As shown in **Figure 6**, protein–protein interactions were largely concentrated in genes between ALI and HUCMSC groups (25). Given the importance of hub genes in the network, we used the plugin cytoHubba to screen hub genes from the PPI network in cytoscape (**Figure 6A**). A subnetwork with three nodes and three edges was identified. Three genes (*Tbx2*, *Nkx2-1*, and *Atf5*) were shown to play a key role in ALI. Notably, the expression of *Tbx2* and *Nkx2-1* was decreased, whereas that of *Atf5* was increased in ALI rats. The four sets of hub genes were subjected to GO/KEGG/and pathway enrichment analysis (**Supplementary Figures 6A–C**). The results of the analysis show that the biological processes and KEGG pathways affected by the core genes are linked in different groups (**Figures 6B–D** **)**, such as, calcium ion transmembrane transport, nuclear division, non membrane bounded organelle assembly, calcium signaling pathway and immune respose etc (**Figures 6E, F**).

## Discussion

MSCs have received marked attention from scientific and clinical researchers in recent years owing to their unique immune regulatory functions. To date, many clinical studies have been conducted to investigate the therapeutic potential of MSCs (26–28). The safety and efficacy of MSC therapy has been confirmed in phase I, II, and III clinical trials for various pathologies including graft-versus-host disease, lupus erythematosus, systemic scleroderma, and rheumatoid arthritis. Notably, preclinical studies support the initial localization of MSCs in the lungs. Indeed, other studies (29–32) have identified that MSC-derived items help ameliorate ALI and pulmonary fibrosis. In Shiao-Ya Hong’s experiments, 1 × 10^5^, 5 × 10^5^, or 1 × 10^6^ allogenic hUC-MSCs were intravenously injected to treat ALI C57BL/6 mice induced by LPS and only partial ALI repair was observed (19). To better explore the effect of HUMSCs on ALI, we repeatedly administered LPS and a 0.4 × 10^6^ cells/kg dose of HUCMSC to rats at 2-day intervals based our primary study on effect of different dosages in HUCMSC-treated ALI rats (data not shown). Notably, RAN-Seq technology was applied to explore the signaling pathways and the possible mechanisms involved in ALI treatment of HUCMSC for the first time. In this study, we identified the benefits of HUCMSC treatment in reducing the severity of LPS-induced ALI in SD rats. After HUCMSC treatment, we observed a substantial decrease in alveolar exudates, total protein content, and proinflammatory factors, and an improvement in lung ventilation and alveolar integrity.

Using RNA-Seq, we summarized the expression patterns and conducted bioinformatics analysis of protein-coding genes that may participate in the pathogenesis of ALI. We identified 308 DEGs by screening [p < 0.05, |log2 (fold change) | > 1] and confirmed 24 candidate genes associated with ALI: *CXCL2*, *CXCL1*, *CXCL6*, *IL10*, *LIF*, *IL12RB2*, *IL22*, *NFKBIA*, *IFNG*, *IL12A*, *IL6*, *BIRC2*, *IL17A*, *IL17C*, *IL17F*, *CXCL12*, *IL1R1*, *TRADD*, *MMP9*, *CCND1*, *IL19*, *FADD*, *BCL2A1*, and *TNFAIP3*. To elucidate the role and mechanisms of these DEGs, GO and KEGG pathway analyses were conducted, which revealed a close relationship of HUCMSC with the inflammatory response, developmental process, and positive modulation of cellular processes.

Currently, multiple signaling pathways have been found to be closely related to ALI, including the NF-κB (33), Hippo–YAP (34), JAK/STAT, and MAPK signaling pathways (35–37). Our KEGG results demonstrated that HUCMSC can regulate ALI-related signaling pathways. Furthermore, our GO analysis revealed that HUCMSC could counteract the abnormal expression of 745 DEGs associated with inflammation, such as adaptive and innate immune responses and IL-6 production genes. These results indicate that HUCMSC decrease the inflammatory response of ALI through regulating these signaling pathways and the immune system, including immune cell activation and immune factor secretion.

Some studies (38, 39) have suggested that excessive accumulation of ECM contributes to pulmonary fibrosis. After lung injury, TGF-β promotes wound repair by increasing ECM production and deposition, inflammatory cell recruitment, and fibroblast production (40). Excessive ECM accumulation and abnormal lung repair lead to tissue scarring, distorted alveolar architecture, and irreversible loss of lung function, ultimately leading to respiratory failure and death (41). In our experiments, genes related to ECM organization and assembly increased expression in the lung tissue of ALI rats but decreased after treatment with HUCMSC. Consistent with previous studies, our results suggest that excessive ECM deposition in LPS-induced ALI was prevented by HUCMSC, inhibiting the progression of pulmonary fibrosis.

Our DEG analysis results demonstrated that the expression of the 297 DEGs associated with lung function, such as oxygen transportation, muscle contraction, and muscle development, was suppressed in ALI but promoted after HUCMSC treatment. This data is consistent with the HUCMSC transcript profile, suggesting improvement of pulmonary ventilation function. Furthermore, our PPI and hub analysis results showed that *TBX21*, *NKX2-1*, and *ATF5* may play key roles in the pathogenesis of ALI, which has not been previously reported (42–46). Our data suggest that HUCMSC may improve ALI symptoms by regulating *TBX21*, *NKX2-1*, and *ATF5* expression and their related pathways.

In conclusion, we determined the role of HUCMSC in the treatment of LPS-induced ALI using RNA-Seq data. Our results indicated that HUCMSC have a therapeutic effect on LPS-induced ALI. HUCMSC inhibit inflammation and pulmonary fibrosis and improve lung ventilation function by preventing damage. Our findings suggest that the targets of HUCMSC are a series of immune cells, cytokines and signaling pathways. In addition, this study is the first to report the potential roles of *TBX21*, *NKX2-1*, and *ATF5* in ALI, which may be important targets for HUCMSC. This study provides new insights into the mechanism of HUCMSC in ALI treatment, and the results can provide experimental evidence that supports further investigation of HUCMSC in the treatment of ALI.

## Data availability statement

The data presented in the study are deposited in the Sequence Read Archive repository, accession Number SRR21074410, SRR21074408, SRR21074409.

## Ethics statement

The studies involving human participants were reviewed and approved by the ethics committee of S−Evans Biosciences (no. 2020−01). The patients/participants provided their written informed consent to participate in this study. All experiments were conducted according to the protocols approved by the local Medical Animal Experiment Ethics Committee.

## Author contributions

EC, LwZ and XP designed the study, conducted animal experiment. QZ and LZ wrote the manuscript. FfW, NC and LL collected the samples, performed blood gas analysis, BALF and histological detection. WC and HC analyzed the RNA-Sequencing. AL and FW performed experiments of qPCR and data analysis. JL and RP supervised the experiment process and revised the paper. All authors contributed to the article and approved the submitted version.

## Funding

This work was supported by the Key Technologies R&D Program of Zhejiang Province (grant number 2019C03041, 2021C03077); and the Creative Research Program of Yuhang (grant number 2020YK009).

## Conflict of interest

Authors XP, QZ, LZ, FfW and HC were employed by S-Evans Biosciences.

The remaining authors declare that the research was conducted in the absence of any commercial or financial relationships that could be construed as a potential conflict of interest.

## Publisher’s note

All claims expressed in this article are solely those of the authors and do not necessarily represent those of their affiliated organizations, or those of the publisher, the editors and the reviewers. Any product that may be evaluated in this article, or claim that may be made by its manufacturer, is not guaranteed or endorsed by the publisher.

## Supplementary material

Supplementary Figure 1 HUCMSC was characterized by morphology, differentiation capacity and surface markers. (A) The morphology of HUCMSC. (B) Differentiation ability test. Osteogenic, adipogenic, and chondrogenic differentiation capacity were detected using Alizarin Red, Oil Red O, and Alcian Blue staining, respectively. Scale bar = 50 μm. (C) Phenotypic analysis. Surface markers were detected by flow cytometry.

Supplementary Figure 2 Transcriptome data analysis. (A) Percent of reads mapped to genome regions. The green part refers to percentage of reads mapped to CDS; the light pink part refers to percentage of reads mapped to intergenic regions; the salmon part refers to percentage of reads mapped to introns. (B) Transcriptome data quality control information statistics table of each sample.

Supplementary Figure 3 The data normalization. (A-C) The Scatter plot of gene comparison between A vs B, C vs B and C vs A. Left: using an ordinary log2(X+1) transformation. Middle: Using the rlog transformation. Right: Using the vst (variance-stabilizing) transformation. (D) The baxplot showed that the sample integrity of the three group samples is high. A, normal group; B, LPS-induced ALI group; C, LPS-induced ALI group with HUCMSC treatment. n=3.

Supplementary Figure 4 Analysis of the overlap of genera and GO terms related with IL-6 regulation. (A) Venn diagrams shows the intersection of GO enrichment of genes in 6 clusters (i). The histogram shows the number of Go terms in each cluster (ii). (B) Cnetplot showing the correlation between intersect genes and the GO terms which were related with the regulation of IL-6. Black hollow circles indicate the size of the GO (BP) terms. Each color represents a cluster. Genes of significant intersection are simply marked in red.

Supplementary Figure 5 MA plots showing genes with a BH-adjusted *P*-value below 0.05 and Log2 fold change (FC) above 0.585 plotted by Log2FC in the y-axis and Log2 mean expression in the x-axis. Upregulated genes are colored in red and downregulated genes are colored in blue. Non-significant genes are colored in gray and denoted as NS. (A) MA plot of top hub genes between normal and ALI. (B) MA plot of top hub genes between ALI and HUCMSC. (C) The qPCR results indicate the up- and down-regulated hub genes marked in color in the above A panel. (D) The qPCR results indicate the up- and down-regulated hub genes marked in color in the above B panel. n=3. *, *P* < 0.05, **, *P* < 0.01.

Supplementary Figure 6 The GO/KEGG/Pathway enrichment analysis results of hub genes from PPI screening. (A) GO enrichment analysis result of hub genes between groups. (B) KEGG enrichment analysis result of hub genes between groups. (C) Enrich pathway analysis result of hub genes between groups. Adjusted *P* value represents significance of terms; GeneRatio represents the ratio of the number of hub genes targets in this GO/KEGG/Pathway term to the number of hub genes targets in all GO/KEGG/Pathway term. GO, gene ontology; KEGG, Kyoto Encyclopedia of Genes and Genomes; Pathway, pathway analysis based on REACTOME pathway database.
