# Supplementary material for: RNA-Sequencing approach for exploring the therapeutic effect of umbilical cord mesenchymal stem/stromal cells on lipopolysaccharide-induced acute lung injury
Source: Front Immunol. 2022 Oct 20;13:1021102. doi: 10.3389/fimmu.2022.1021102 (PMC9632738; doi:10.3389/fimmu.2022.1021102)
Supplement: Supplementary file 5 [file Table_1.docx]

**Supplementary table 1**The intersecting different genes of comparing groups

| B vs A DN \| C vs B DN | B vs A DN \| C vs B Up | B vs A Up \| C vs B DN | B vs A Up \| C vs B Up |
| --- | --- | --- | --- |
| MSTRG.10291 | ENSRNOG00000008176 | ENSRNOG00000043445 | ENSRNOG00000060703 |
| MSTRG.20608 | MSTRG.13939 | ENSRNOG00000026919 | ENSRNOG00000010253 |
| MSTRG.14916 | ENSRNOG00000005555 | ENSRNOG00000046005 | ENSRNOG00000013727 |
| MSTRG.5815 | MSTRG.120 | ENSRNOG00000046192 | ENSRNOG00000014960 |
| ENSRNOG00000013962 | ENSRNOG00000008141 | ENSRNOG00000022309 | ENSRNOG00000018770 |
| MSTRG.20267 | ENSRNOG00000046295 | ENSRNOG00000003280 | ENSRNOG00000013862 |
| ENSRNOG00000046667 | MSTRG.13937 | ENSRNOG00000001242 | ENSRNOG00000022537 |
| ENSRNOG00000017084 | ENSRNOG00000004583 | ENSRNOG00000021039 | ENSRNOG00000031431 |
| MSTRG.20726 | ENSRNOG00000007600 | ENSRNOG00000012134 | ENSRNOG00000014336 |
| ENSRNOG00000019296 | MSTRG.5296 | ENSRNOG00000019504 | ENSRNOG00000011654 |
| ENSRNOG00000013408 | ENSRNOG00000058323 | ENSRNOG00000046890 | ENSRNOG00000010018 |
| ENSRNOG00000010938 | MSTRG.5151 | ENSRNOG00000056290 | ENSRNOG00000047314 |
| ENSRNOG00000006033 | MSTRG.1561 | ENSRNOG00000049024 | ENSRNOG00000009339 |
| ENSRNOG00000019361 | ENSRNOG00000051734 | MSTRG.5585 | ENSRNOG00000000239 |
| ENSRNOG00000037080 | ENSRNOG00000060604 | MSTRG.13561 | ENSRNOG00000016561 |
| ENSRNOG00000038319 | ENSRNOG00000017833 | ENSRNOG00000042319 | ENSRNOG00000046699 |
| MSTRG.2557 | ENSRNOG00000050888 | MSTRG.4770 | ENSRNOG00000003208 |
| ENSRNOG00000059507 | ENSRNOG00000014815 | ENSRNOG00000031229 | ENSRNOG00000015465 |
| ENSRNOG00000018853 | ENSRNOG00000020837 | ENSRNOG00000047599 | ENSRNOG00000020281 |
| ENSRNOG00000047951 | ENSRNOG00000014409 | ENSRNOG00000056052 | ENSRNOG00000005115 |
| ENSRNOG00000026941 | ENSRNOG00000028945 | MSTRG.4688 | ENSRNOG00000028993 |
|  | ENSRNOG00000025757 | ENSRNOG00000008478 | ENSRNOG00000028137 |
|  | ENSRNOG00000014327 | MSTRG.230 | ENSRNOG00000007387 |
|  | ENSRNOG00000012307 | MSTRG.12112 | MSTRG.8775 |
|  | ENSRNOG00000018250 | ENSRNOG00000013593 | ENSRNOG00000037302 |
|  | ENSRNOG00000050675 | MSTRG.21128 | ENSRNOG00000018815 |
|  | ENSRNOG00000055450 | ENSRNOG00000049870 | ENSRNOG00000019048 |
|  | ENSRNOG00000012303 | MSTRG.5000 | ENSRNOG00000006198 |
|  | ENSRNOG00000038625 |  | ENSRNOG00000008759 |
|  | ENSRNOG00000016837 |  | ENSRNOG00000049115 |
|  | MSTRG.9181 |  | ENSRNOG00000020991 |
|  | ENSRNOG00000056179 |  | ENSRNOG00000028415 |
|  | MSTRG.5860 |  | ENSRNOG00000012749 |
|  | ENSRNOG00000009466 |  | ENSRNOG00000004487 |
|  | ENSRNOG00000033734 |  | ENSRNOG00000004682 |
|  | ENSRNOG00000018943 |  | ENSRNOG00000048411 |
|  | ENSRNOG00000012477 |  | ENSRNOG00000008165 |
|  | ENSRNOG00000005269 |  | ENSRNOG00000008575 |
|  | ENSRNOG00000019851 |  | ENSRNOG00000049033 |
|  | ENSRNOG00000011754 |  | ENSRNOG00000009785 |
|  | ENSRNOG00000023227 |  | ENSRNOG00000053272 |
|  | ENSRNOG00000020719 |  | ENSRNOG00000002723 |
|  | ENSRNOG00000016243 |  | ENSRNOG00000001414 |
|  | ENSRNOG00000016437 |  | ENSRNOG00000015423 |
|  | MSTRG.18027 |  | ENSRNOG00000053047 |
|  | ENSRNOG00000057927 |  | ENSRNOG00000029115 |
|  | ENSRNOG00000012609 |  | ENSRNOG00000061595 |
|  | ENSRNOG00000023803 |  | ENSRNOG00000004921 |
|  | ENSRNOG00000010268 |  | ENSRNOG00000015520 |
|  | ENSRNOG00000019183 |  | ENSRNOG00000058539 |
|  | ENSRNOG00000007461 |  | ENSRNOG00000015514 |
|  | MSTRG.4592 |  | ENSRNOG00000019100 |
|  | ENSRNOG00000023943 |  | ENSRNOG00000012835 |
|  | ENSRNOG00000028965 |  | ENSRNOG00000011777 |
|  | MSTRG.17859 |  | ENSRNOG00000013598 |
|  | ENSRNOG00000060486 |  | MSTRG.517 |
|  | ENSRNOG00000002607 |  | ENSRNOG00000027655 |
|  | ENSRNOG00000020997 |  | ENSRNOG00000008736 |
|  | ENSRNOG00000013605 |  | ENSRNOG00000007159 |
|  | MSTRG.18580 |  | ENSRNOG00000015131 |
|  | ENSRNOG00000058068 |  | ENSRNOG00000027811 |
|  | ENSRNOG00000005659 |  | ENSRNOG00000009334 |
|  | ENSRNOG00000061299 |  | ENSRNOG00000008588 |
|  | ENSRNOG00000012557 |  | ENSRNOG00000030187 |
|  | ENSRNOG00000020369 |  | ENSRNOG00000010210 |
|  | ENSRNOG00000028814 |  | ENSRNOG00000046834 |
|  | ENSRNOG00000000871 |  | ENSRNOG00000020953 |
|  | ENSRNOG00000021802 |  | ENSRNOG00000016535 |
|  | ENSRNOG00000003888 |  | ENSRNOG00000026235 |
|  | ENSRNOG00000023410 |  | ENSRNOG00000036834 |
|  | MSTRG.17889 |  | ENSRNOG00000019890 |
|  | MSTRG.20302 |  | ENSRNOG00000004393 |
|  | MSTRG.1761 |  | ENSRNOG00000027855 |
|  | ENSRNOG00000057092 |  | ENSRNOG00000014378 |
|  | ENSRNOG00000019162 |  | ENSRNOG00000009919 |
|  | ENSRNOG00000026762 |  | ENSRNOG00000007886 |
|  | ENSRNOG00000031576 |  |  |
|  | MSTRG.1173 |  |  |
|  | ENSRNOG00000045924 |  |  |
