# Supplementary figures and images for: RNA-Sequencing approach for exploring the therapeutic effect of umbilical cord mesenchymal stem/stromal cells on lipopolysaccharide-induced acute lung injury
Source: Front Immunol. 2022 Oct 20;13:1021102. doi: 10.3389/fimmu.2022.1021102 (PMC9632738; doi:10.3389/fimmu.2022.1021102)

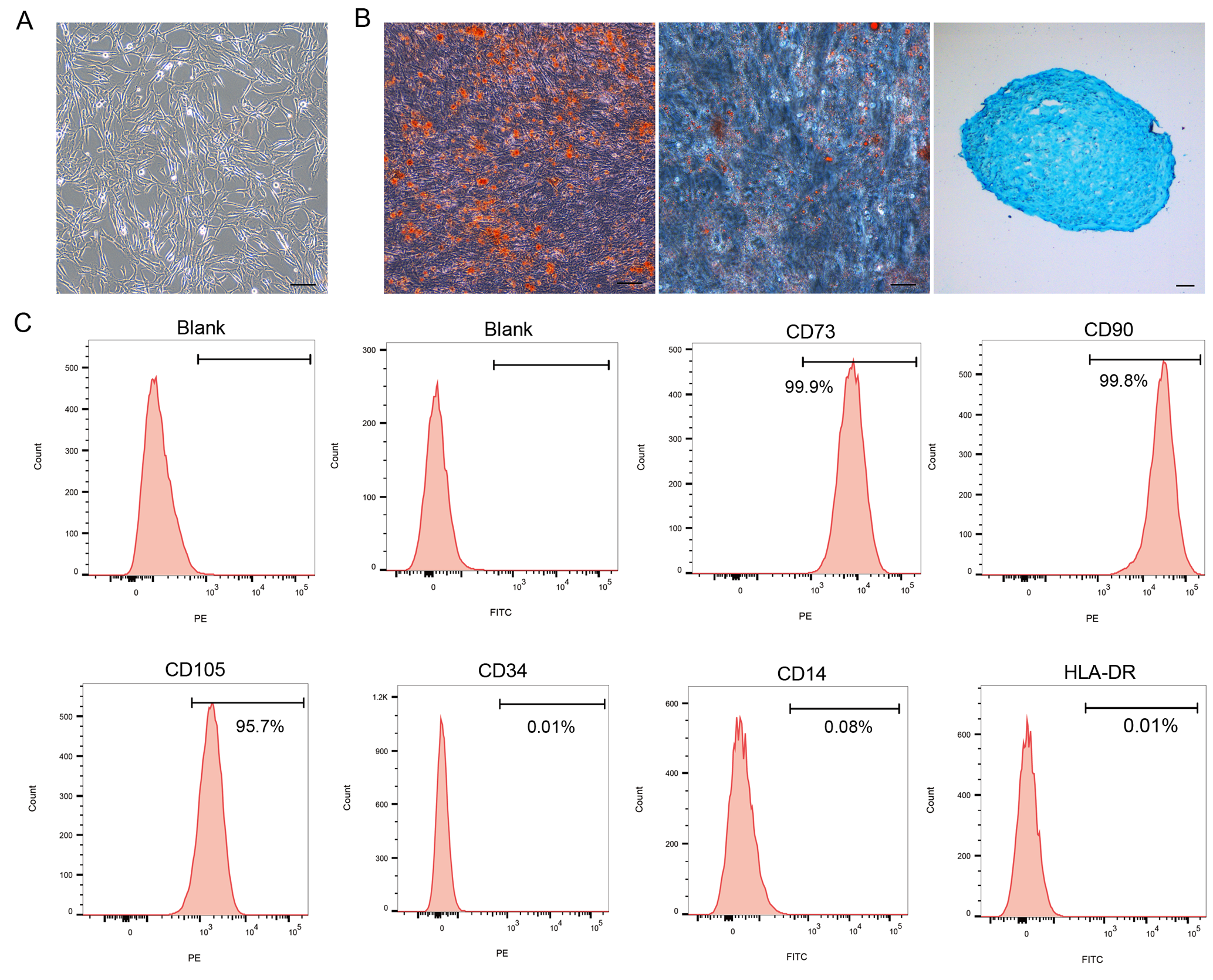

Supplement: Supplementary Figure 1 — HUCMSC was characterized by morphology, differentiation capacity and surface markers. (A) The morphology of HUCMSC. (B) Differentiation ability test. Osteogenic, adipogenic, and chondrogenic differentiation capacity were detected using Alizarin Red, Oil Red O, and Alcian Blue staining, respectively. Scale bar = 50 μm. (C) Phenotypic analysis. Surface markers were detected by flow cytometry. [file Image_1.tif]

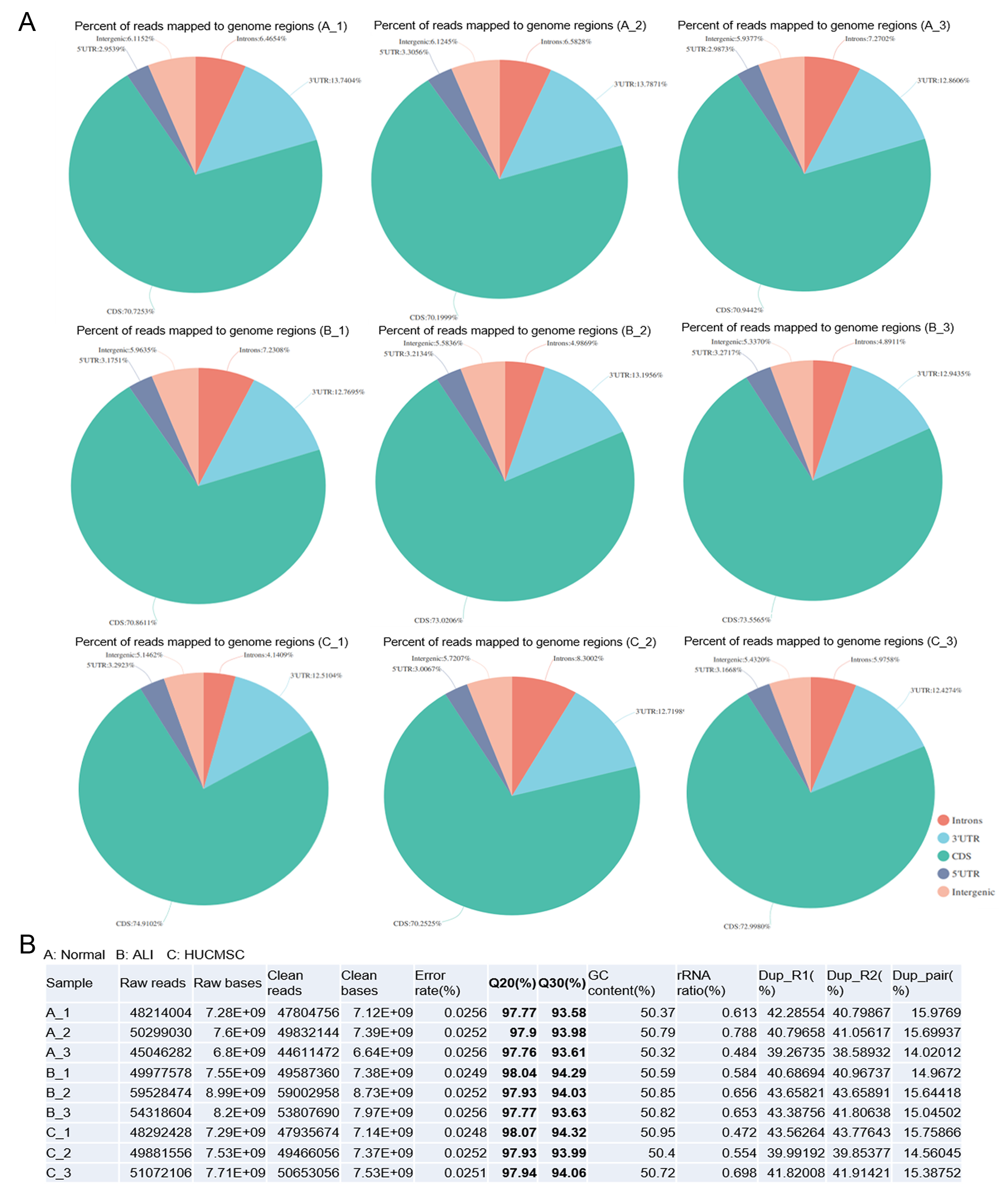

Supplement: Supplementary Figure 2 — Transcriptome data analysis. (A) Percent of reads mapped to genome regions. The green part refers to percentage of reads mapped to CDS; the light pink part refers to percentage of reads mapped to intergenic regions; the salmon part refers to percentage of reads mapped to introns. (B) Transcriptome data quality control information statistics table of each sample. [file Image_2.tif]

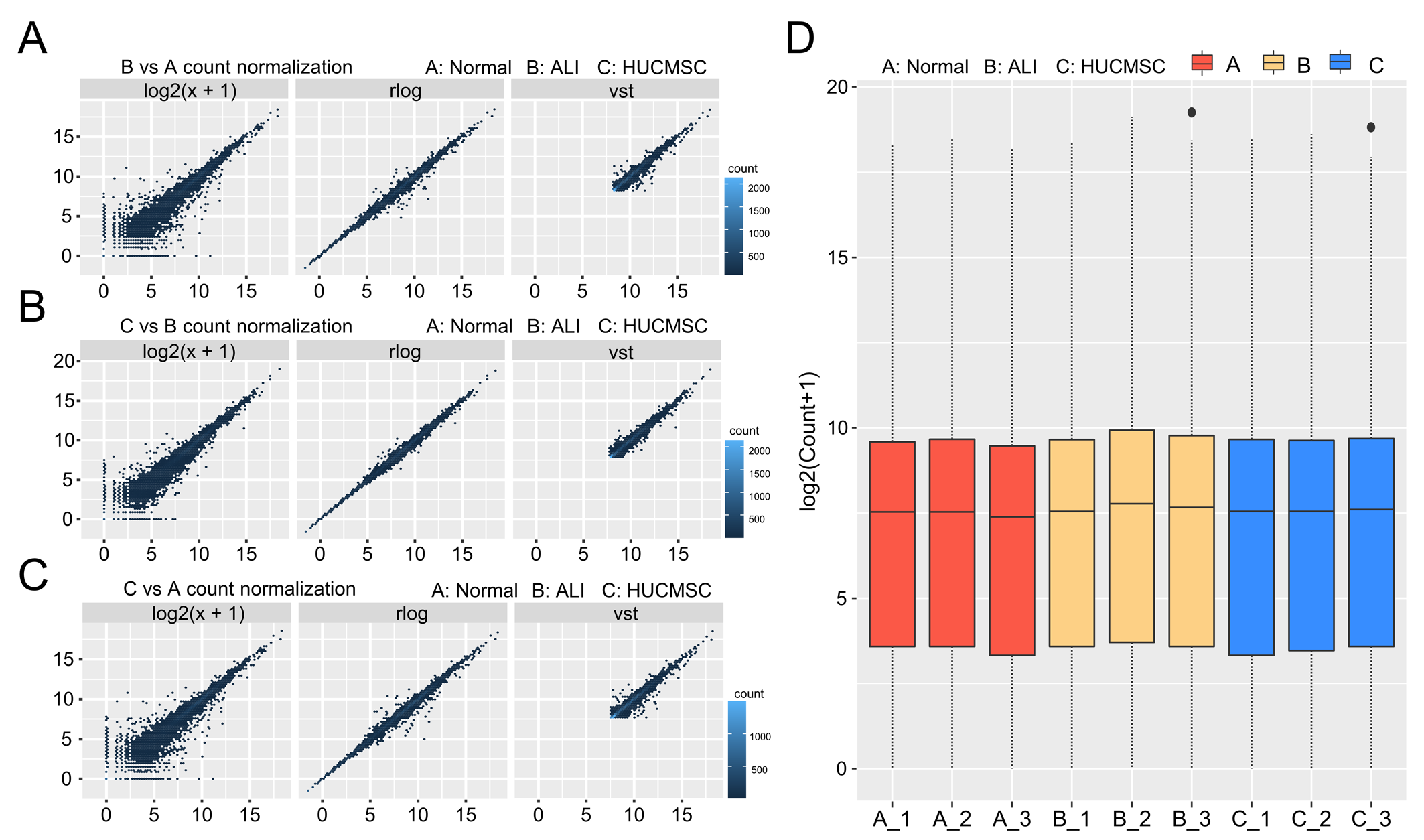

Supplement: Supplementary Figure 3 — The data normalization. (A-C) The Scatter plot of gene comparison between A vs B, C vs B and C vs A. Left: using an ordinary log2(X+1) transformation. Middle: Using the rlog transformation. Right: Using the vst (variance-stabilizing) transformation. (D) The baxplot showed that the sample integrity of the three group samples is high. A, normal group; B, LPS-induced ALI group; C, LPS-induced ALI group with HUCMSC treatment. n=3. [file Image_3.tif]

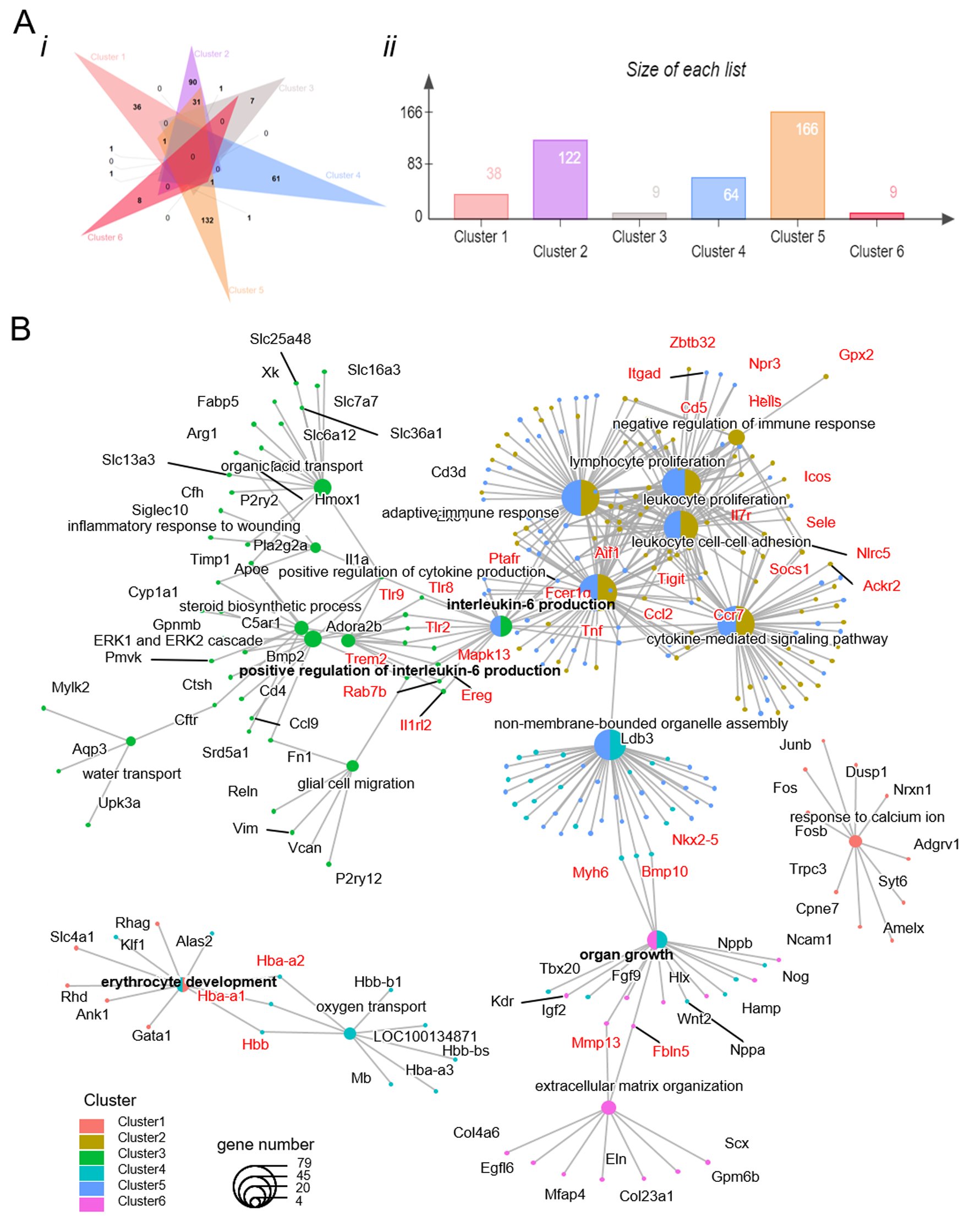

Supplement: Supplementary Figure 4 — Analysis of the overlap of genera and GO terms related with IL-6 regulation. (A) Venn diagrams shows the intersection of GO enrichment of genes in 6 clusters (i). The histogram shows the number of Go terms in each cluster (ii). (B) Cnetplot showing the correlation between intersect genes and the GO terms which were related with the regulation of IL-6. Black hollow circles indicate the size of the GO (BP) terms. Each color represents a cluster. Genes of significant intersection are simply marked in red. [file Image_4.tif]

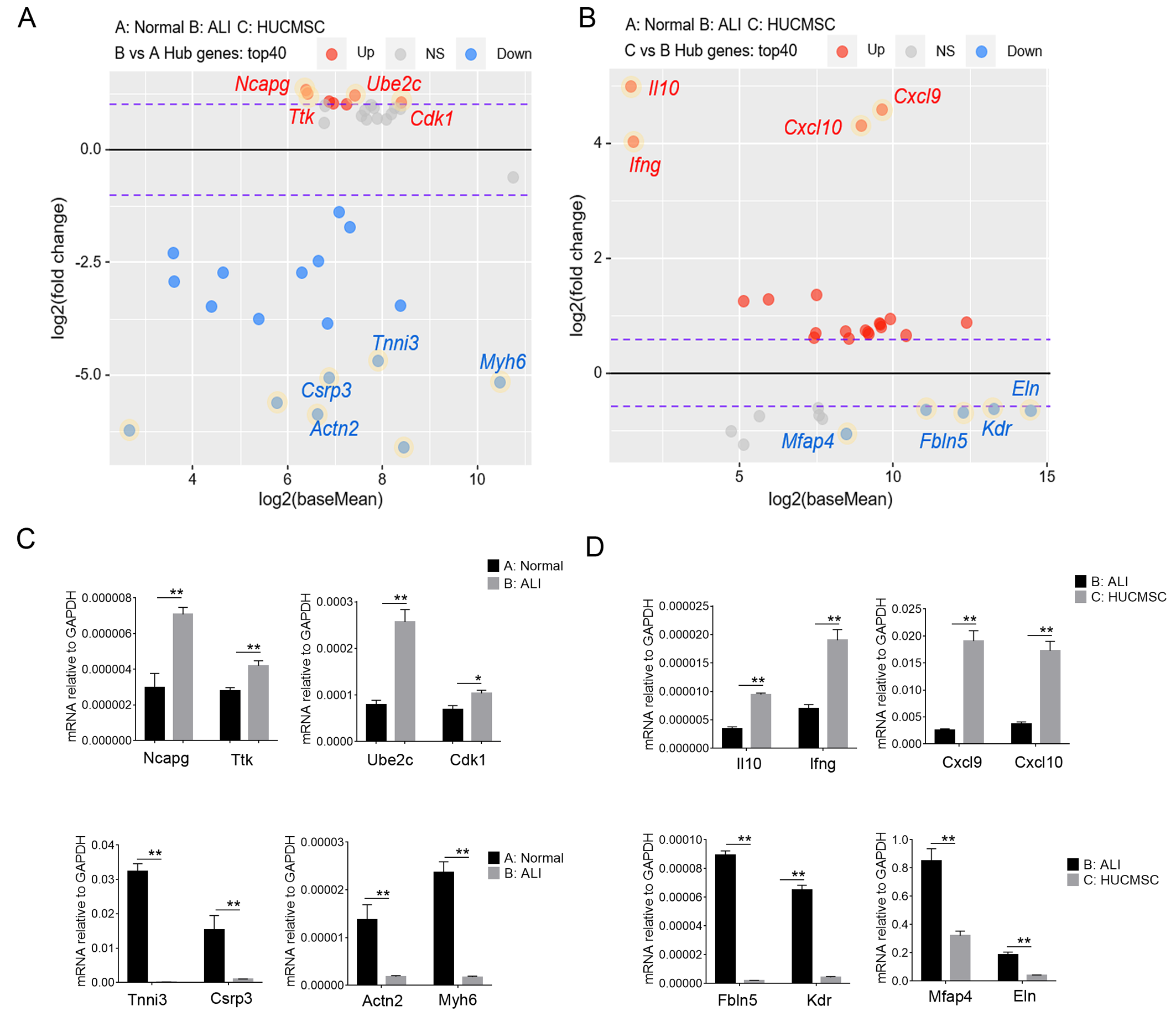

Supplement: Supplementary Figure 5 — MA plots showing genes with a BH-adjusted P-value below 0.05 and Log2 fold change (FC) above 0.585 plotted by Log2FC in the y-axis and Log2 mean expression in the x-axis. Upregulated genes are colored in red and downregulated genes are colored in blue. Non-significant genes are colored in gray and denoted as NS. (A) MA plot of top hub genes between normal and ALI. (B) MA plot of top hub genes between ALI and HUCMSC. (C) The qPCR results indicate the up- and down-regulated hub genes marked in color in the above A panel. (D) The qPCR results indicate the up- and down-regulated hub genes marked in color in the above B panel. n=3. *, P < 0.05, **, P < 0.01. [file Image_5.tif]

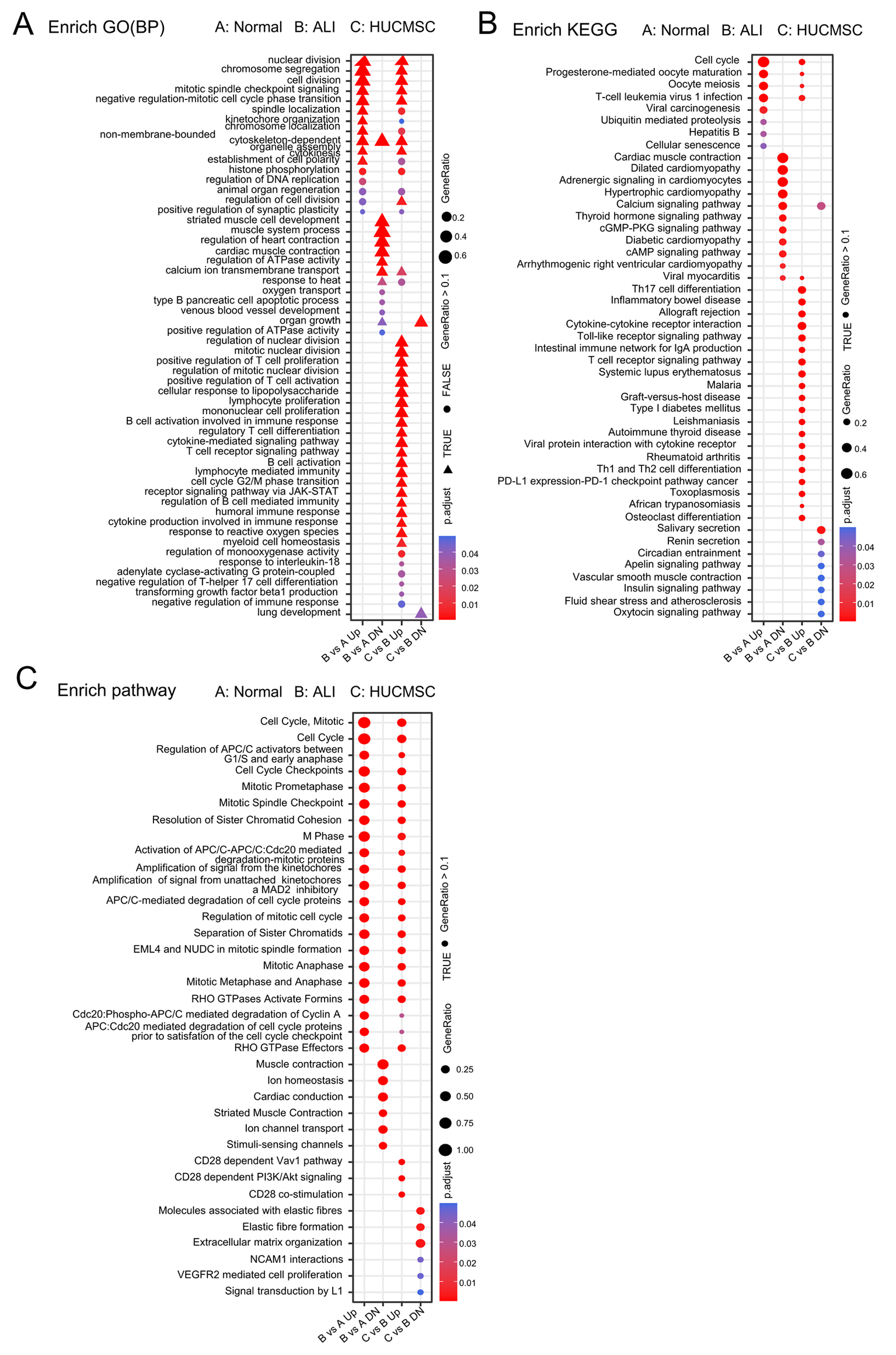

Supplement: Supplementary Figure 6 — The GO/KEGG/Pathway enrichment analysis results of hub genes from PPI screening. (A) GO enrichment analysis result of hub genes between groups. (B) KEGG enrichment analysis result of hub genes between groups. (C) Enrich pathway analysis result of hub genes between groups. Adjusted P value represents significance of terms; GeneRatio represents the ratio of the number of hub genes targets in this GO/KEGG/Pathway term to the number of hub genes targets in all GO/KEGG/Pathway term. GO, gene ontology; KEGG, Kyoto Encyclopedia of Genes and Genomes; Pathway, pathway analysis based on REACTOME pathway database. [file Image_6.tif]
